# Salud mental en la población bogotana: análisis de la Encuesta Nacional de Salud Mental

**DOI:** 10.15446/rsap.V25n3.93116

**Published:** 2023-05-01

**Authors:** Felipe Botero-Rodríguez, Catalina López-Figueroa, Daniela Yucumá, Andrés Salgado-Cendales, Juan P. Acevedo-Gallego, Julián G. Rodríguez-Barrios, Liliana González-Cabrales, Santiago Bolívar-Moná, Carlos J. Rincón, Carlos Gómez-Restrepo

**Affiliations:** 1 FB: MD. M. Sc. Epidemiología Clínica. Departamento de Epidemiología Clínica y Bioestadística, Pontificia Universidad Javeriana. Bogotá, Colombia. felipe.botero@javeriana.edu.co Pontificia Universidad Javeriana Departamento de Epidemiología Clínica y Bioestadística Pontificia Universidad Javeriana Bogotá Colombia felipe.botero@javeriana.edu.co; 2 CL: Epidemióloga. Pontificia Universidad Javeriana. Bogotá, Colombia. catalina_lopez@javeriana.edu.co Pontificia Universidad Javeriana Pontificia Universidad Javeriana Bogotá Colombia catalina_lopez@javeriana.edu.co; 3 DY: MD. Pontificia Universidad Javeriana. Bogotá, Colombia. yucumad@javeriana.edu.co Pontificia Universidad Javeriana Pontificia Universidad Javeriana Bogotá Colombia yucumad@javeriana.edu.co; 4 AS: Epidemiólogo. Pontificia Universidad Javeriana, Bogotá, Colombia. andres_salgado@javeriana.edu.co Pontificia Universidad Javeriana Pontificia Universidad Javeriana Bogotá Colombia andres_salgado@javeriana.edu.co; 5 JA: Epidemiólogo. Pontificia Universidad Javeriana. Bogotá, Colombia. juan_acevedo@javeriana.edu.co Pontificia Universidad Javeriana Pontificia Universidad Javeriana Bogotá Colombia juan_acevedo@javeriana.edu.co; 6 JR: Epidemiólogo. Pontificia Universidad Javeriana. Bogotá, Colombia. jg.rodriguez@javeriana.edu.co Pontificia Universidad Javeriana Pontificia Universidad Javeriana Bogotá Colombia jg.rodriguez@javeriana.edu.co; 7 LG: Epidemiologa. Pontificia Universidad Javeriana. Bogotá, Colombia. liliana_gonzalez@javeriana.edu.co Pontificia Universidad Javeriana Pontificia Universidad Javeriana Bogotá Colombia liliana_gonzalez@javeriana.edu.co; 8 SB: Epidemiólogo. Pontificia Universidad Javeriana. Bogotá, Colombia. santiago_bolivar@javeriana.edu.co. Pontificia Universidad Javeriana Pontificia Universidad Javeriana Bogotá Colombia santiago_bolivar@javeriana.edu.co; 9 CR: Estadístico. M. Sc. Epidemiología Clínica. M. Sc. Bioestadística. Pontificia Universidad Javeriana. Bogotá, Colombia. carlosrincon@javeriana.edu.co Pontificia Universidad Javeriana Epidemiología Clínica Pontificia Universidad Javeriana Bogotá Colombia carlosrincon@javeriana.edu.co; 10 CG: MD. Psiquiatra. M. Sc. Epidemiología Clínica. Ph.D. Salud Pública. Pontificia Universidad Javeriana. Bogotá, Colombia. cgomez@javeriana.edu.co Pontificia Universidad Javeriana Epidemiología Clínica Salud Pública Pontificia Universidad Javeriana Bogotá Colombia cgomez@javeriana.edu.co

**Keywords:** Salud mental, trastornos mentales, trastornos relacionados con sustancias, encuestas epidemiológicas, salud pública *(fuente: DeCS, BIREME)*, Mental health, mental disorders, substance-related disorders, health surveys, public health *(source: MeSH, NLM)*

## Abstract

**Objetivos:**

Caracterizar la salud mental de la población de Bogotá (Colombia), a partir de información de la última Encuesta Nacional de Salud Mental (2015).

**Metodología:**

Analizamos cuantitativamente la información de población residente en Bogotá recolectada en la Encuesta Nacional de Salud Mental (2015) para calcular la prevalencia de problemas y trastornos de salud mental, así como de consumo de sustancias psicoactivas con sus intervalos de confianza del 95 %. El análisis se estratificó por grupos etarios.

**Resultados:**

Encontramos lo siguiente: a) un autorreporte de problemas de salud mental del 12 % (IC95 %: 9,1-15,6) y 7,9 % (IC95 %: 5,3-11,8) para los adultos y adolescentes encuestados, respectivamente; b) 9,8 % (IC95 %: 7,4-12,9) y 4,6 % (IC95 %: 2,7-7,7) con alto número de síntomas de ansiedad en adultos y adolescentes, respectivamente; c) presencia de un número alto de síntomas depresivos en el 6,7 % (IC95 %: 4,9-9,0) y 3,5 % (IC95 %: 2,0-6,1) de la población de adultos y adolescentes respectivamente; d) 12,9 % (IC95 %: 10 %-16,4 %) de los adultos y el 8,5 % (IC95 %: 5,5 %-12,8 %) de los adolescentes había presentado "cualquier trastorno de salud mental" en la vida; e) consumo de alcohol en algún momento de la vida en el 43,8 % (IC95 %: 39,1-48,7) y consumo de marihuana en un 5,6 % (IC95 %: 3,7-8,5) alguna vez en la vida.

**Conclusiones:**

La población bogotana reporta prevalencias distintas a las de otros lugares del país y de otras capitales en la región. Esta caracterización permitirá guiar el desarrollo de políticas de prevención y mitigación en la ciudad, teniendo en cuenta que Bogotá agrupa un gran porcentaje de población de diversos orígenes y culturas.

## Abstract (en)

**Objectives:**

To Characterize the population of Bogotá (Colombia), in terms of mental health, based on data from the 2015-National Mental Health Survey.

**Metodology:**

A quantitative analysis was employed. Data for population living in Bogotá and collected in the 2015-National Mental Health Survey was employed to calculate the prevalence of mental health problems, disorders, and the use of psychoactive substances. The analysis was stratified in adults, adolescents, and children.

**Results:**

Our main findings were a) The prevalence of self-reported mental health problems was 12 % (95 % CI: 9.1-15.6) and 7.9 % (95 % CI: 5.3-11, 8) for adults and adolescents, respectively. b) 9.8 % (95 % CI: 7.4-12.9) of included adults and 4.6 % (95 % CI: 2.7-7.7) of the adolescents reported more than 5 symptoms of anxiety. c) 6.7 % (95 % CI: 4.9-9.0) and 3.5 % (95 % CI: 2.0-6.1) of the adult and adolescent reported more than 7 symptoms of depression. d) 12.9 % (95 % CI: 10 % - 16.4 %) of the included adults, and 8.5 % (95 % CI: 5.5 % - 12.8 %) of adolescents reported the presence of "any mental health disorder" in the curse of life. e) 43.8 % (95 % CI: 39.1-48.7) reported consumption of alcohol. 5.6 % (95 % CI: 3.7-8.5) reported to have consumed marijuana during their life.

**Conclusions:**

The prevalence of mental health problems and disorders is different as compared to other regions of the country and even to other capital cities in Latin America. Therefore, this characterization could guide mental health decisions, mostly because Bogotá groups population with diverse origins and cultures.

En Colombia el interés por la salud pública en términos de salud mental (SM) ha crecido considerablemente en los últimos años debido a un aumento de la carga de enfermedad de entidades neuropsiquiátricas 1,4, de los años de vida potencialmente perdidos por suicidio y de la prevalencia del consumo problemático de alcohol y otras sustancias psicoactivas 5. En el año 2010 las enfermedades mentales, en su conjunto, fueron la primera causa de años vividos con discapacidad y representaron el 7,4 % de años de vida ajustados por discapacidad 6. Dentro de las aproximaciones más recientes a esta problemática en Colombia, se encuentra la Encuesta Nacional de Salud Mental (ENSM) realizada en 2015 7.

De otro lado, Bogotá es una de las capitales en América Latina que agrupa mayor número de habitantes y culturas. Se estima para 2025 su población superará los 10 millones de habitantes 8. En otras megaciudades de la región, como Sao Paulo y Ciudad de México, el aumento de la población ha provocado a su vez un aumento de las alteraciones de SM. Además, las proyecciones demográficas estiman un aumento de la población concentrada en megaciudades localizadas particularmente en países en vía de desarrollo 9. Sin embargo, las caracterizaciones en términos de SM de la población de capitales como bogotana son escasas. Contar con esta información es relevante, máxime al considerar que la SM se relaciona con factores sociales y ambientales que pueden alterarse con el crecimiento demográfico de alguna región 10.

En este trabajo caracterizamos a la población bogotana con base en la información de la ENSM 2015 y en los principales factores asociados a la sintomatología reportada. Esto se realizó con el propósito de aportarles información relevante a quienes toman decisiones, a fin de contribuir a que direccionen sus esfuerzos teniendo en cuenta la promoción y la prevención de la salud mental en Bogotá.

## METODOLOGÍA

Esta investigación se realizó a partir de la información de población residente en Bogotá, no institucionalizada, recolectada en la Encuesta Nacional de Salud Mental (ENSM) 2015. Esta es una encuesta aplicada a una submuestra de la muestra maestra para estudios poblacionales en salud, diseñada por el Ministerio de Salud y Protección Social de Colombia, cuyos resultados tienen representatividad a regional y local. Esta muestra fue de tipo probabilístico, polietápica y estratificada por sexo y edad 7.

En el estudio se incluyó toda la población residente de Bogotá seleccionada aleatoriamente y consintió en participar para responder la encuesta. Se utilizaron instrumentos de tamizaje para problemas de salud mental, síntomas de estrés postraumático, consumo de alcohol y sustancias psicoactivas, disfunción familiar y trastornos mentales 4,11. Basado en ello, las variables incluidas en nuestro estudio fueron divididas en datos sociodemográficos, problemas en salud mental, síntomas de trastornos en salud mental y consumo de sustancias psicoactivas.

Para el análisis, los datos fueron ponderados por medio de factores de expansión de la muestra, teniendo en cuenta la estimación de las varianzas en encuestas complejas a través del método de linealización mediante series de Taylor; se presentan las prevalencias expresadas en valores absolutos y porcentajes con sus intervalos de confianza del 95 % (IC95 %) estratificado por grupos etarios, en adultos (>17 años), adolescentes (11-17 años) y niños (<11 años).

## RESULTADOS

Fueron entrevistados 2 451 individuos, de los cuales 1 735 eran adultos, 300 eran adolescentes y 416, niños. La mayoría eran mujeres y residían en zona urbana. En general, no se reportó disfunción familiar por parte de los adultos (89,6 %), similar a los adolescentes (88 %). Respecto al nivel de educación de la población, la mayoría de los adultos completaron hasta la secundaria; algunos, la primaria, y otros ningún ciclo terminado, educación universitaria o educación tecnológica; en el caso de los adolescentes, la mayoría respondieron al nivel educativo completado como primaria o ninguno, y en el grupo de niños, el 98,2 % se encontró en proceso de escolarización (Tabla 1).

**Tabla 1:** Características sociodemográficas de la población

Variables sociodemográficas		n	%	IC95 %	
Adultos	Edad				
	adultos 18 a 44	845	59,3	54,7	63,8
	adultos 45 o más	881	40,7	36,2	45,3
	Estado civil				
	Casado-UL-pareja	885	52,9	48,1	57,6
	Separado/viudo/divorciado	380	13,8	11,1	17,1
	Soltero	461	33,3	28,9	38,1
	Pobreza				
	Hogares en estado de pobreza	89	4,2	3,2	5,6
	Nivel educativo				
	Ninguno/primaria	460	21,7	17,9	26,0
	Secundaria	925	56,7	51,9	61,4
	Técnico/tecnológico	169	10,4	7,7	13,8
	Universitario	150	11,2	8,6	14,5
	Sexo				
	Hombre	645	48,4	43,6	53,2
	Mujer	1081	51,6	46,8	56,4
	Usted se reconoce como...				
	Gitano/ROM	1	0,0	0,0	0,2
	Indígena	66	2,6	1,9	3,7
	Negro, mulato, afrodescendiente o afrocolombiano	65	4,2	2,5	6,9
	Raizal del archipiélago de San Andrés y Providencia	1	0,0	0,0	0,0
	Ninguno de los anteriores	1593	93,1	90,4	95,1
	Zona urbana/rural				
	Rural	45	4,4	2,9	6,8
	Urbana	1681	95,6	93,2	97,1
	Disfunción familiar				
	Personas sin disf. familiar	1475	89,6	87,0	91,8
	Personas con disf. familiar leve	136	6,9	5,1	9,4
	Personas con disf. familiar moderada	58	1,5	1,0	2,2
	Personas con disf. familiar severa	57	1,9	1,3	3,0
Adolescentes	Edad				
	12	50	16,2	10,3	24,5
	13	49	16,2	9,5	26,4
	14	50	17,8	12,2	25,3
	15	51	19,9	11,6	31,9
	16	46	13,9	9,6	19,7
	17	54	16,0	11,2	22,4
	Pobreza				
	Hogares en estado de pobreza	32	12,0	7,5	18,5
	Nivel educativo				
	Ninguno/primaria	188	62,8	52,6	72,0
	Secundaria	109	36,0	26,9	46,3
	Técnico/tecnológico	3	1,2	0,3	4,8
	Sexo Hombre	145	48,4	38,8	58,1
	Mujer	155	51,6	41,9	61,2
	Usted se reconoce como.				
	Indígena	5	1,1	0,4	2,8
	Negro, mulato, afrodescendiente o afrocolombiano	11	3,2	1,7	6,1
	Ninguno de los anteriores	284	95,7	92,7	97,5
	Zona urbana/rural				
	Rural	6	1,0	0,4	2,8
	Urbana	294	99,0	97,2	99,6
	Disfunción familiar				
	Personas sin disf. familiar	255	88,0	82,5	91,9
	Personas con disf. familiar leve	33	9,2	5,8	14,4
	Personas con disf. familiar moderada	5	0,9	0,4	2,3
	Personas con disf. familiar severa	7	1,9	0,8	4,5
Niños	Edad				
	7	88	18,2	13,2	24,6
	8	89	24,4	17,7	32,6
	9	67	16,9	10,4	26,2
	10	79	18,6	13,4	25,3
	11	93	21,9	15,9	29,4
	Escolarizado				
	Si	408	98,2	95,8	99,3
	Pobreza				
	Hogares en estado de pobreza	34	6,6	4,3	10,0
	Sexo				
	Hombre	231	48,4	40,1	56,7
	Mujer	185	51,6	43,3	59,9
	El niño se reconoce como... Indígena	6	0,7	0,3	1,7
	Negro, mulato, afrodescendiente o afrocolombiano	15	4,0	1,6	9,9
	Ninguno de los anteriores	395	95,3	89,8	97,9
	Zona urbana/rural				
	Rural	15	3,2	1,8	5,8
	Urbana	401	96,8	94,2	98,2

Disf.: disfunción.

### Problemas de salud mental

El 12 % (IC95 %: 9,1-15,6) de los adultos encuestados presentó algún problema en salud mental (evaluado con el Self-Reporting Questionnaire [SRQ]); mientras que, en la población adolescente, esta proporción fue de 7,9 % (IC95 %: 5,3-11,8). Respecto a sintomatología específica, el 9,8 % (IC95 %: 7,4-12,9) y el 4,6 % (IC95 %: 2,7-7,7) presentaron un número de síntomas alto para ansiedad (más de 5) para adultos y adolescentes, respectivamente. La presencia de un número alto de síntomas depresivos (más de 7) se observó en el 6,7 % (IC95 %: 4,9-9,0) y 3,5 % (IC95 %: 2,0-6,1) de la población. En cuanto a síntomas psicóticos (uno de dos síntomas indicadores), hubo presencia en el 8 % de los adultos y en el 6 % de los adolescentes. Adicionalmente, la vivencia de al menos un evento traumático se presentó en el 49,4 % y en el 16,45 % de adultos y adolescentes respectivamente. En los niños, según el Report Questionnaire for Children (RQC), el 46,6 %, el 38,2 % y el 10,7 % hubo 0,1 y dos o más problemas de salud mental (Tabla 2).

**Tabla 2:** Prevalencia de problemas en salud mental

	Problemas de salud mental	n	%	IC95 %	
Adultos	Ansiedad*				
	Ningún síntoma	740	44,3	39,5	49,2
	Número de síntomas bajo de ansiedad (1 a 2 síntomas)	566	33,5	29,2	38,1
	Número de síntomas medio de ansiedad (3 a 4 síntomas)	247	12,5	10,1	15,3
	Número de síntomas alto de ansiedad (más de 5 síntomas)	173	9,8	7,4	12,9
	Depresión*				
	Número de síntomas bajo de depresión (1 a 3 síntomas)	1269	75,2	71,2	78,7
	Número de síntomas medio de depresión (4 a 6 síntomas)	326	18,2	15,1	21,8
	Número de síntomas alto de depresión (más de 7 síntomas)	131	6,7	4,9	9,0
	Epilepsia*				
	No	1702	98,8	97,9	99,3
	Un síntoma indicador sugestivo de epilepsia positivo	24	1,2	0,7	2,2
	Global*				
	negativo	1536	88,0	84,4	90,9
	positivo	190	12,0	9,1	15,6
	Psicosis*				
	1 de 2 síntomas indicadores de psicosis positivo	125	8,0	5,7	11,2
	No	1601	92,0	88,8	94,3
	Total de eventos traumáticos por persona				
	No han sufrido ningún evento traumático	831	50,6	45,8	55,4
	Un evento traumático	530	26,9	23,4	30,8
	Dos eventos traumáticos	162	9,8	7,5	12,8
	Tres eventos traumáticos	88	6,6	4,2	10,2
	Cuatro o más eventos traumáticos	82	6,0	4,0	9,1
	Síntomas de estrés postraumático				
	Sí	40	2,9	1,9	4,6
Adolescentes	Ansiedad*				
	Ningún síntoma	143	50,2	40,5	60,0
	Número de síntomas bajo de ansiedad (1 a 2 síntomas)	111	38,1	28,9	48,3
	Número de síntomas medio de ansiedad (3 a 4 síntomas)	28	7,0	4,4	11,0
	Número de síntomas alto de ansiedad (más de 5 síntomas)	18	4,6	2,7	7,7
	Depresión*				
	Número de síntomas bajo de depresión (1 a 3 síntomas)	232	81,5	74,9	86,6
	Número de síntomas medio de depresión (4 a 6 síntomas)	52	15,0	10,4	21,3
	Número de síntomas alto de depresión (más de 7 síntomas)	16	3,5	2,0	6,1
	Epilepsia*				
	No	296	99,1	97,3	99,7
	Un síntoma indicador sugestivo de epilepsia positivo	4	0,9	0,3	2,7
	Global*				
	negativo	268	92,1	88,2	94,7
	positivo	32	7,9	5,3	11,8
	Psicosis*				
	1 de 2 síntomas indicadores de psicosis positivo	25	6,0	3,8	9,4
	No	275	94,0	90,6	96,2
	Total de eventos traumáticos por persona				
	No han sufrido ningún evento traumático	186	65,8	57,3	73,4
	Un evento traumático	75	22,4	16,4	29,8
	Dos eventos traumáticos	25	7,6	4,8	11,9
	Tres eventos traumáticos	12	3,6	1,7	7,4
	Cuatro o más eventos traumáticos	2	0,6	0,1	2,3
	Síntomas de estrés postraumático	4	2,5	0,9	7.1
	Sí				
Niños	Problemas de salud mental en población infantil**				
	No tiene problemas	200	46,6	38,3	55,1
	Tiene un problema	144	38,2	30,2	46,9
	Tiene dos o más problemas	72	15,2	10,7	21,2
	Total de eventos traumáticos				
	No han sufrido ningún evento traumático	347	83,5	76,6	88,7
	Un evento traumático	60	15,5	10,4	22,5
	Dos, tres, cuatro o más eventos traumáticos	9	1,0	0,5	1,9
	Síntomas de estrés postraumático				
	Sí	9	9,7	3,7	23,0

*SRQ; **RQC; IC95 %: Intervalo de confianza del 95 %.

### Trastornos de salud mental

Según los criterios de la American Psychiatric Association (APA), el 12,9 % (IC95 %: 10 %-16,4 %) de los adultos, y el 8,5 % (IC95 %: 5,5 %-12,8 %) de los adolescentes habían presentado "cualquier trastorno de salud mental" en la vida. Al indagar por cada trastorno, se encontraron prevalencias de vida de 10,7 (IC95 %: 8,0 %-14,2 %) y de 2,2 % (IC95 %: 1,2 % 4,3 %) para trastornos del afecto en adultos y adolescentes respectivamente. Por el lado de ansiedad, la prevalencia fue de 5,0 % (IC95 %: 3,5 % 7,2 %) y 7,0 % (IC95 %: 4,3 % 11,2 %). Mientras que el intento de suicidio se reportó en el 4,6 % (IC95 %: 2,9 7,2) de adultos y en el 0,9 % (IC95 %: 0,3-2,7) de los adolescentes. En los niños, el 5 % (IC95 %: 2,1 11,5) presentó cualquier trastorno en el último año (Tabla 3).

**Tabla 3:** Prevalencia de trastornos en salud mental

	Trastornos de Salud Mental	n	%	IC95 %	
Adultos	Intento de suicidio	61	4,6	2,9	7,2
	Plan suicida	55	4,5	2,6	7,8
	Ideación suicida	148	10,3	7,6	13,8
	DSM-IV Afecto (12 meses)	62	3,4	2,1	5,4
	DSM-IV Afecto (30 días)	12	0,4	0,2	0,7
	DSM-IV Afecto (alguna vez en la vida)	192	10,7	8,0	14,2
	DSM-IV Ansiedad (12 meses)	50	2,3	1,5	3,4
	DSM-IV Ansiedad (30 días)	26	1,0	0,6	1,8
	DSM-IV Ansiedad (alguna vez en la vida)	94	5,0	3,5	7,2
	DSM-IV Cualquier trastorno (12 meses)	96	4,9	3,4	7,0
	DSM-IV Cualquier trastorno (30 días)	37	1,4	0,9	2,2
	DSM-IV Cualquier trastorno (alguna vez en la vida)	240	12,9	10,0	16,4
Adolescentes	Intento de suicidio	4	0,9	0,3	2,7
	Plan suicida	2	1,2	0,3	5,8
	Ideación suicida	16	5,6	3,2	9,7
	DSM-IV Afecto (12 meses)	7	1,3	0,6	3,0
	DSM-IV Afecto (30 días)	3	0,3	0,1	1,0
	DSM-IV Afecto (alguna vez en la vida)	11	2,2	1,2	4,3
	DSM-IV Ansiedad (12 meses)	16	3,7	2,0	6,8
	DSM-IV Ansiedad (30 días)	11	2,2	1,1	4,6
	DSM-IV Ansiedad (alguna vez en la vida)	29	7,0	4,3	11,2
	DSM-IV Cualquier trastorno (12 meses)	20	4,5	2,6	7,7
	DSM-IV Cualquier trastorno (30 días)	14	2,6	1,3	4,9
	DSM-IV Cualquier trastorno (alguna vez en la vida)	36	8,5	5,5	12,8
Niños	DISC (último 12 meses) - Afecto	2	0,2	0,0	0,6
	DISC (último 12 meses) - Algún trastorno de inicio de la infancia	14	4,3	1,6	11,1
	DISC (último 12 meses) - Ansiedad	7	0,9	0,4	2,1
	DISC (último 12 meses) - Cualquier trastorno	19	5,0	2,1	11,5
	DISC (último mes) - Afecto	1	0,1	0,0	0,6
	DISC (último mes) - Algún trastorno de inicio de la infancia	11	3,7	1,2	10,9
	DISC (último mes) - Ansiedad	5	0,7	0,3	1,8
	DISC (último mes) - Cualquier trastorno	14	4,1	1,4	11,1

DSM: Diagnostic and Statistical Manual of Mental Disorders; DISC: Diagnostic Interview Schedule for Children; IC 95%: intervalo de confianza del 95 %.

#### Consumo de sustancias psicoactivas

El alcohol fue la sustancia con mayor consumo en la vida (43,8 %; IC95 %: 39,1-48,7) de los cuales la mayoría consumió en los últimos 12 meses (79,3 %). Respecto a problemas relacionados con su consumo, el 9,7 % (IC95 %: 7,2-12,9) presentó consumo excesivo y el 12,2 % (IC95 %: 10,2-14,7) eran bebedores en riesgo. En cuanto al cigarrillo, el 5 % (IC95 3,7-6,8) lo ha probado alguna vez en la vida, y de ellos, el 7,2 lo consumió en el último año. La marihuana fue la sustancia ilícita de mayor consumo, con un 5,6 % (IC95 %: 3,7-8,5), de los cuales 4,3 % habían consumido el último año. Finalmente, en cuanto a otras sustancias, la prevalencia de consumo alguna vez en la vida fue de 3,2 % (IC95 %: 1,5-6,4) (6 % los últimos 12 meses). De la población encuestada, el 40 % (IC95 %: 35,6-44,6) reportó no haber consumido ninguna de las sustancias mencionadas.

En los adolescentes, el alcohol también tuvo la mayor prevalencia de consumo (27 %; IC95 %: 18,8-38,5 en la vida), de los cuales el 86,1 % consumió en los últimos 12 meses; sin embargo, hubo menor proporción de consumo excesivo o riesgoso (2,2 % y 3,6 % respectivamente). La segunda sustancia con mayor prevalencia de consumo fue la marihuana (3,9 % la ha consumido alguna vez en su vida; de ellos 4,8 % [IC95 %: 1,6-13,3] la consumió en el último año). El 1,4 % (IC95 %: 0,7-3) de los encuestados reportó consumo de alguna otra sustancia psicoactiva, mientras que el 0,8 % (IC95 %: 0,2-3,3) reportó consumo de cigarrillo. Por otro lado, el 66,2 % (IC95 %: 55,8-75,3) de los adolescentes encuestados no ha consumido ninguna de estas sustancias (Tabla 4).

**Tabla 4:** Prevalencia de consumo de sustancias psicoactivas

	Sustancias psicoactivas	n	%	IC95 %	
Adultos	Consumo de sustancias alguna vez en la vida				
	Alcohol	687	43,8	39,1	48,7
	Cigarrillo	102	5,0	3,7	6,8
	Marihuana	79	5,6	3,7	8,5
	Otras	65	5,5	3,2	9,4
	Ninguna de las anteriores	793	40,0	35,6	44,6
	Consumo de sustancias en los últimos 12 meses				
	Alcohol	670	79,3	73,1	84,4
	Cigarrillo	77	7,2	5,0	10,2
	Marihuana	20	4,3	1,4	12,3
	Otras	18	3,2	1,5	6,4
	Ninguna de las anteriores	73	6,0	4,1	8,7
	Problemas relacionados con consumo de alcohol				
	Probable dependencia al alcohol	10	0,5	0,2	1,1
	Consumo excesivo	144	9,7	7,2	12,9
	Bebedores en riesgo	228	12,2	10,1	14,7
	Ninguno de los anteriores	1344	77,7	73,9	81,0
Adolescentes	Consumo de sustancias alguna vez en la vida				
	Alcohol	76	27,6	18,8	38,5
	Cigarrillo	2	0,8	0,2	3,3
	Marihuana	13	3,9	1,9	7,9
	Otras	8	1,4	0,7	3,0
	Ninguna de las anteriores	201	66,2	55,8	75,3
	Consumo de sustancias en los últimos 12 meses				
	Alcohol	73	86,1	72,5	93,5
	Cigarrillo	1	3,6	0,5	21,7
	Marihuana	6	4,8	1,6	13,3
	Otras	4	2,8	0,9	8,1
	Ninguna de las anteriores	2	2,8	0,7	10,9
	Problemas relacionados con consumo de alcohol				
	Consumo excesivo	8	2,2	0,9	5,1
	Bebedores en riesgo	11	3,6	1,6	7,8
	Ninguno de los anteriores	281	94,2	89,7	96,8

IC 95%: intervalo de confianza del 95 %.

## DISCUSIÓN

Los temas relacionados con la SM han tenido un creciente interés debido al aumento de su prevalencia y la consecuente carga de enfermedad 1,2. Su importancia se refleja en el desarrollo de políticas públicas regionales y nacionales 12. Sin embargo, no encontramos en la literatura una caracterización específica sobre la SM de la población bogotana. Por tal razón, en este trabajo caracterizamos a la población en términos de SM a partir de información de la más reciente ENSM, con el propósito de aportar información a los tomadores de decisiones, que permita direccionar los esfuerzos en términos de promoción y prevención de la SM en Bogotá.

### Hallazgos en salud mental

Bogotá cuenta con características distintas a las demás ciudades y municipios del país. Por ser la capital del país, concentra la mayor cantidad y diversidad de personas y culturas, así como diversas realidades y vivencias. Teniendo en cuenta esto, identificamos que la prevalencia general de problemas de salud mental (y específicamente la presencia de síntomas de ansiedad y depresión o de al menos un síntoma psicótico en la población adulta) se encuentra por encima de las correspondientes prevalencias acumuladas en el país. Esta situación es similar a la de la proporción de personas que han vivido un evento traumático; al respecto, la población bogotana se encuentra por encima del resto del país, con excepción de Antioquia 7.

A pesar de que el autorreporte de algún problema en salud mental (12 %) es mayor respecto al resto de país, sigue siendo menor la prevalencia en comparación con otras ciudades como Sao Paulo, Brasil (19,7 %)^13^. No obstante, debemos ser cautelosos al realizar estas comparaciones, pues cada estudio emplea muestras con criterios de selección y puntos de corte distintos para determinar dichas prevalencias, además de las considerables diferencias demográficas entre las ciudades.

Con respecto a los trastornos de salud mental, las prevalencias de alguno de estos disminuyó en comparación con la información consignada en la ENSM de 2003 14. Para el 2015, encontramos una prevalencia de trastornos de ansiedad del 5 %, de afecto del 10,7 % o de cualquier trastorno, del 12,9 % (sin incluir adicción a sustancias psicoactivas ni control de impulsos) 7; mientras que en reportes más antiguos (2003), las prevalencias reportadas fueron de 10 %, 6,8 % y 17,8 % (incluyendo adicción a sustancias psicoactivas, control de impulsos y los anteriores) respectivamente 15.

Las cifras mencionadas cobran importancia al señalar un aumento de la tasa de mortalidad por trastornos mentales y del comportamiento en la última década 16. No obstante, reportamos una disminución en trastornos específicos, como de cualquier trastorno de ansiedad, que pasó de 21,7 % a 5 % entre 2003 y 2015 7,14. Este descenso se da a pesar de ser la ansiedad uno de los trastornos más prevalentes en el mundo, con comparadores como Distrito Federal en Brasil, con una prevalencia de 6,69 % en 2007 y una prevalencia global de 3,86 % para el mismo año 17.

Esta tendencia no fue diferente en los adolescentes; de hecho, el problema más prevalente fue el de ansiedad (por lo menos alguna vez en la vida). En estudios previos que evalúan la epidemiología de las enfermedades de salud mental en adolescentes y niños a nivel mundial, como el de Merikangas et ál. 18, se reporta una prevalencia de ansiedad cercana al 8 %; en el presente estudio, por ejemplo, la prevalencia es del 7 %. El estudio de Llorca et ál. 19 considera que una posible explicación es que este grupo etario no cuenta con mecanismos adaptativos suficientes para enfrentar los problemas y superar los retos que trae esta etapa de la vida. Este puede llegar a ser un factor de riesgo de ansiedad para aquellos adolescentes que no cuenten con un adecuado soporte.

En cuanto a los trastornos del afecto, la literatura reporta una prevalencia cercana al 4 % (0,2-17 %) en adolescentes 18, lo cual dista de lo encontrado en nuestro estudio (2,2 %). Sin embargo, la baja tasa en nuestra población podría asociarse a barreras en el acceso a los servicios de salud identificadas en la ENSM 2015, que reportó como limitación principal las barreras actitudinales, que corresponden a bajo interés en asistir a las citas o miedo debido a la estigmatización y poco conocimiento de estas patologías 7.

Estas diferencias en las prevalencias también podrían ser explicadas por diversos aspectos metodológicos (distintos instrumentos, diferentes definiciones y distintas poblaciones) y en las diferencias culturales, como el hecho de estigmatizar los problemas de salud mental, lo cual ocasiona un subregistro de estos trastornos 20. También podrían deberse a la presencia de otros factores asociados como pertenecer a hogares de menores ingresos económicos o tener una familia disfuncional 21.

Finalmente, los niveles de ideación suicida en la ENSM 2015 fueron bajos en comparación con los resultados de un estudio realizado por la OMS en 32 países de ingresos medios y bajos. El estudio de la OMS reportó una prevalencia de ideación suicida de entre 5,1 % y 28,1 % e ideación con plan entre 1,7 % y 15,3 %. Esto refleja la heterogeneidad de este índice entre países y la complejidad del análisis de estas variables dada la multiplicidad de factores asociados 22.

### Consumo de sustancias psicoactivas

Según el Estudio de Consumo de Sustancias Psicoactivas en Bogotá 23, el 89,46 % de la población encuestada entre 12 y 65 años reportó consumo de alcohol alguna vez en la vida, lo cual contrasta con el 43,8 % y 27,6 % en adultos y adolescentes, respectivamente, según la ENSM 2015 7. Por grupo etario, se encontró una mayor prevalencia en el consumo de alcohol entre adolescentes bogotanos, en comparación con los adolescentes de todo el territorio nacional, con un 27,6 % y 19,32 %, respectivamente, cifras elevadas por un posible consumo naturalizado de alcohol 24.

En cuanto al consumo de marihuana, se ha estimado una prevalencia del 13,37 % en población de 12 a 65 años 20, mientras que en la ENSM 2015 es del 5,6 % (IC95 %: 3,5-9,5) y 3,9 % (IC95 %: 1,9-7,9) en adultos y adolescentes, respectivamente. Por otra parte, el consumo de tabaco entre adolescentes de Bogotá en comparación con Colombia es menor, con prevalencias de 0,8 % en Bogotá, y de 4,77 % en Colombia 23. Respecto a la percepción de riesgo por consumir estas sustancias, la percepción de alto riesgo al fumar es menor en adolescentes, sobre todo al cambiar su presentación como ocurre con los sistemas electrónicos de administración de nicotina, aunque esta percepción aumenta con la edad 23,25,26. En contraste, la percepción de alto riesgo por consumo de marihuana es mayor: alcanza un 58,3 % de los encuestados en el Estudio de Consumo de Sustancias Psicoactivas. No obstante, la mayoría de la población considera que es fácil conseguir cualquiera de estas sustancias 20.

En este estudio encontramos prevalencias distintas a las reportadas en otros lugares del país e incluso a nivel mundial. Debido a las particularidades de la población bogotana, es destacable la importancia de caracterizar a la población de forma particular, en especial si se tiene en cuenta que agrupa un gran porcentaje de población de diversos orígenes y culturas. El conocimiento sobre las características particulares de salud mental de la población es fundamental para informar el desarrollo de políticas y medidas de prevención y mitigación en la ciudad. Finalmente, sigue siendo necesario ampliar el conocimiento en esta área, en particular en ciudades colombianas con grandes grupos poblacionales, en las que aún existe poca literatura sobre la caracterización, en términos de salud mental, de la población ♦
